# Amelioration of Ovalbumin-Induced Food Allergy in Mice by Targeted Rectal and Colonic Delivery of Cyanidin-3-O-Glucoside

**DOI:** 10.3390/foods11111542

**Published:** 2022-05-24

**Authors:** Jie Li, Chao Zou, Yixiang Liu

**Affiliations:** 1 College of Ocean Food and Biological Engineering, Jimei University, Xiamen 361021, China; shijiexiaoa@163.com; 2 Gaoan Public Inspection and Testing Center, Gao’an 330800, China; zouchaoknight@163.com; 3 Collaborative Innovation Center of Provincial and Ministerial Co-Construction for Marine Food Deep Processing, Dalian Polytechnic University, Dalian 116034, China

**Keywords:** cyanidin-3-O-glucoside, food allergy, ovalbumin, intestinal barrier, intestinal microbiome

## Abstract

Targeted rectal and colonic delivery is an effective strategy to exploit the biological functions of polyphenols. This work investigated the anti-food allergy (FA) activity of cyanidin-3-O-glucoside (C3G) delivered by enteric sodium alginate in vivo. The results showed that through targeted rectal and colonic delivery, the C3G showed better results in ameliorating clinical allergic symptoms, diarrhea, and serological indicators including ovalbumin-specific IgE, histamine, and mast cell protease-1. The C3G was more efficient in enhancing the intestinal epithelial barrier by up-regulating the tight junction protein expression and promoting secretory IgA and β-defensin secretion. The improved bioactivity in regulating T helper (Th)1/Th2 immune balance in the intestinal mucosa was also observed. Compared with the intestinal microbiota structure of the model group, targeted rectal and colonic delivery of C3G was able to bring the abundance of *Bacteroidota* and *Firmicutes* close to the levels found in normal mice. Furthermore, there was an evident increase in beneficial bacteria in the intestinal flora, such as *Lactobacillus* and *Odoribacter*, and a decrease in pathogenic bacteria like *Helicobacter* and *Turicibacter*. Therefore, the anti-FA activity of C3G could be increased via targeted rectal and colonic delivery, while the mechanism might be attributed to the regulation of intestinal microecological homeostasis.

## 1. Introduction

Food allergy (FA), defined as “an adverse health effect arising from a specific immune response that occurs reproducibly on exposure to a given food,” is an increasingly prevalent health concern worldwide [1,2]. It is estimated that nearly 5% of adults and 8% of children are affected by FAs [3]. There is compelling evidence that dietary polyphenols hold promise as anti-allergy agents capable of influencing multiple biological pathways and immune cell functions during the allergic immune response [1]. However, polyphenolic compounds, especially anthocyanins, are susceptible to structural decomposition in the gastrointestinal tract, severely limiting their biological activity [4]. Recent studies have emphasized that intestinal ecological imbalance, including intestinal immune homeostasis and bacterial structure imbalance, plays a vital role in the occurrence and development of FA [5,6]. Therefore, to improve the anti-FA bioactivity of dietary polyphenols, they must reach the lower gastrointestinal tract intact to fully regulate intestinal ecological balance.

Polysaccharide-based encapsulation can effectively achieve the rectal or colonic delivery of dietary polyphenols. These biological macromolecules can provide a protective barrier for dietary polyphenols, safeguarding their chemical structures and biological activity against damage in the upper gastrointestinal tract [4,7]. It is believed that chitosan-based encapsulation can effectively protect anthocyanins against degradation and improve their antioxidant activity in an in vitro gastrointestinal digestive environment [8]. When alginate was used to encapsulate the phenolic extracts derived from *Clitoria ternatea*, their bioaccessibility increased about 1.5-fold after gastrointestinal digestion simulation [9]. In addition, as the delivery carrier of polyphenols, polysaccharides also displayed distinct advantages regarding the controlled release and the synergistic prebiotic effect in the lower gastrointestinal tract [7,10]. Previous studies have shown that polysaccharides loading dietary polyphenols are mainly through non-covalent binding interactions including hydrogen bonding, van der Waals forces, and hydrophobic interactions, which is influenced by the degree of saturation and aggregation, configuration, and molecular size of polysaccharides [10]. This modifiability of binding affinity makes it possible for polysaccharides to achieve the targeted intestinal release of dietary polyphenols. For example, alginate/chitosan-based embedment can achieve the targeted colonic release of rutin and significantly enhance its efficacy in ameliorating inflammatory bowel disease [11]. The therapeutic effect of quercetin, a dietary polyphenol that can effectively reshape intestinal flora, on intestinal inflammation improved after encapsulation with chitosan/xanthan gum [12].

Furthermore, research has demonstrated that FAs are closely related to the intestinal ecological balance [13]. First, the integrity of the intestinal epithelium protects the mucosal immune system from excessive exposure to dietary antigens [8] The tight junction (TJ) proteins among intestinal epithelial cells (IECs) form a physical barrier that prevents unnecessary antigen entry [14]. Additionally, IECs constitutively express the CD23 (Fc epsilon RII) receptor for immunoglobulin E (IgE), enabling the direct presentation of IgE and IgE/antigen complexes in lymphocytes [15]. Second, it is believed that gut-associated intestinal lymphoid tissue represents the largest secondary lymphoid organ in the human body [16]. Normally, this immune tissue can produce a protective immune response to food allergens, known as “oral tolerance” [17]. However, when its immunologic balance is disrupted, oral tolerance fails, leading to FA development [15]. In addition, intestinal microbiota plays a vital role during the early induction and maintenance of oral tolerance to food allergens [18]. For example, colonizing germ-free mice with Clostridial taxa reportedly protected against oral allergenic sensitization [19]. Moreover, beneficial intestinal bacteria can directly stimulate the intestinal immune system to express anti-inflammatory cytokines (e.g., interleukin (IL)-10) and regulate mucosal integrity by producing metabolites, such as short chain fatty acids (SCFAs) [6,18]. Therefore, taking into account the role of gut ecological balance in the regulation of FA and the advantages of polysaccharide-based encapsulation for targeted intestinal delivery of polyphenols mentioned above, it is reasonable to hypothesize that polyphenols retained in higher amounts in the intestine after polysaccharide encapsulation may exert better anti-allergic activity through better regulation of intestinal ecological balance. Our previous study showed that encapsulating cyanidin-3-O-glucoside (C3G) with low-viscosity sodium alginate (LVA) increases bioaccessibility from 46.3% to about 70.0% after gastrointestinal digestion simulation [20]. Alginate-based encapsulation may efficiently deliver C3G to the lower gastrointestinal tract. Therefore, the effect of LVA-based self-assembly on the anti-FA activity of C3G is further investigated in this work. The anti-FA activity of free C3G and LVA-embedded C3G (LVA + C3G complex) was compared using an ovalbumin (OVA)-induced mouse model. Subsequently, the effect of the LVA + C3G complex on intestinal barrier functions and intestinal flora composition is discussed further. This study provides a theoretical basis and technical support for improving FA via the targeted intestinal delivery of dietary functional components.

## 2. Materials and Methods

### 2.1. Regents and Materials 

The C3G (purity > 98%) was acquired from Chengdu Must Bio-Technology Co., Ltd. (Chengdu, China). The sodium alginate with 35–40% of α-L-guluronic acid was purchased from Xilong Chemical Co., Ltd. (Guangzhou, China). The OVA and loratadine (Lora) were obtained from Sigma (St Louis, MO, USA), while the inject alum was purchased from Thermo Fisher Scientific Inc. (Waltham, MA, USA). The histamine, mouse mast cell protease-1 (mMCP-1), and anti-OVA-IgE enzyme-linked immunosorbent assay (ELISA) kits were supplied by IBL (Hamburg, Germany), R&D Systems (Minneapolis, MN, USA), and Cayman (Ann Arbor, MI, USA), respectively. The Na+-K+-ATPase ELISA kit was acquired from the Jiancheng Bioengineering Institute (Nanjing, China). The commercial ELISA kits for interferon (IFN)-γ, tumor necrosis factor (TNF)-α, interleukin (IL)-4, IL-10, IL-18, and IL-22 were obtained from Neobioscience (Shenzhen, China). The mucin-2, β-defense, D-lactate, secretory IgA (sIgA), claudin-1, occludin, and ZO (zonula occludens)-1 were obtained from mlboi (Shanghai, China). BosterBiotech, Inc. (Wuhan, China) supplied the cell lysis buffer and protease inhibitor cocktail. The other analytical-grade reagents were obtained from the China National Pharmaceutical Industry Corporation Ltd. (Shanghai, China).

### 2.2. Preparation of Samples

The LVA + C3G complex was prepared using a self-assembly method based on our previous study [20]. Briefly, 1 g of sodium alginate was dissolved in 50 mL of deionized water. Next, an autoclave reactor (Shanghai Boxun Industry & Commerce Co., Ltd. Medical Equipment Factory, Shanghai, China) was used to degrade the alginate solution at 121 °C and 0.103 MPa for 20 min to obtain low-viscosity sodium alginate (LVA). The C3G was dissolved in hydrochloric acidized water (pH 2.0) to a 2.0 mg/mL concentration. The C3G and LVA solutions (2.0 mg/mL) were pre-cooled at 4 °C in a refrigerator and mixed at an LVA to C3G mass ratio of 3:1. While stirring magnetically, the pH of the reaction system was adjusted to 3 and left to stand for 1 h. The solution was then centrifuged, and the precipitate was lyophilized to obtain the LVA + C3G complex. The LVA + C3G mixture was prepared by directly incorporating C3G into the LVA solution.

### 2.3. Animals

Male BALB/c mice, aged six to eight weeks, were obtained from the Shanghai Laboratory Animal Center of the Chinese Academy of Sciences (Shanghai, China). All mice were exposed to a 12-h dark/light cycle in a clean specific pathogen free room for one week, at temperatures ranging from 22 to 25 °C and humidity levels of 55% to 60%. They were fed a standard breeding diet. All mice used in this study were cared for in accordance with the Guide-lines for the Care and Use of Laboratory Animals published by the US National Institutes of Health (1996), and all experimental procedures were approved by the Institutional Animal Care and Use Committee of the Animal Laboratory Center of Jimei University (Xiamen, China, No. SCXK 2016-0006). In the mouse model, five mice were included in each group.

### 2.4. Protocol of the FA Mouse Model

**Figure 1:**
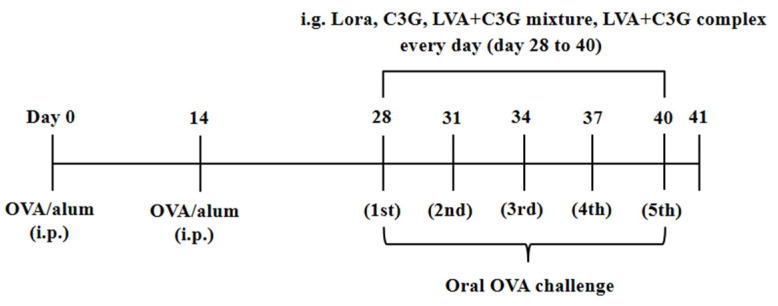
The protocol for the FA model.

As shown in Figure 1, the mice were sensitized and monitored using a method described in previous studies with some modifications [21]. Briefly, mice were sensitized twice with 100 µg OVA and 2.0 mg alum in 200 µL of phosphate-buffered saline (PBS) via intraperitoneal (i.p.) injection at 0 d and 14 d. During the challenge phase two weeks later, the mice received 50 mg OVA (in 200 µL of PBS) five times every 3 d (OVA group). During the OVA challenge phase, the OVA-sensitive and OVA-challenged mice were gavaged once daily with C3G (C3G group), LVA + C3G complex (LVA + C3G complex group), and an LVA + C3G mixture (LVA + C3G mixture group), respectively. The C3G dose was set at 25 mg/kg body weight as described in earlier studies [22], while the LVA + C3G complex and LVA + C3G mixture contained the same C3G dose. Negative control animals (PBS group) were sham-sensitized with alum and challenged with PBS. As a positive control, OVA-sensitized and OVA-challenged mice were intragastrically administered 20 mg/kg of the selective histamine H1 receptor antagonist, Lora (Lora group). Anaphylactic symptoms were evaluated 1 h after each challenge, as previously reported [23]. Anaphylactic symptom scores were assessed as follows: 0—no symptoms; 1—scratching around nose and head; 2—puffiness around eyes and mouth; 3—wheezing, labored respiration, cyanosis around mouth and tail; 4— no activity after prodding or tremors and convulsions; and 5—death. In addition, changes in the rectal temperatures were measured for 1 h after the final challenge. Serum was collected from the tail veins of the mice 1 h after the last challenge, and stored at −80 °C to measure the histamine and mMCP-1 levels [23]. The mice were sacrificed on day 2 after the last OVA challenge, and their tissues and blood were harvested for histological analysis and cytokine determination.

### 2.5. Allergy-Related Factors in the Serum

Serum was obtained after the fifth OVA challenge to measure the allergy-related factors. The OVA-specific IgE, histamine, and mMCP-1 were determined using ELISA kits according to the instructions of the manufacturer.

### 2.6. Histological Examination

The intestinal tissues of the mice were removed at 41 d, fixed in 10% phosphate-buffered formalin, and embedded in paraffin. The paraffin sections (5-μm thick) were subjected to hematoxylin and eosin (H&E) staining and periodic acid-Schiff (PAS) staining. The morphological structures of the intestinal segments, including the intestinal villus height and crypt depth, were evaluated by measuring at least ten different regions in each section using a Nikon 80i (Nikon, Tokyo, Japan) equipped with a biological color microscopy image analysis system. The goblet and PAS-positive cells were counted by analyzing at least 100 epithelial cells in five different intestinal villi in each section from each mouse.

### 2.7. Determination of the Na^+^-K^+^-ATPase Activity 

The Na^+^-K^+^-ATPase activity was assessed by measuring the release of inorganic phosphate (Pi) from ATP according to the ELISA kit protocol (Nanjing Jiancheng Bioengineering Institute, China). Briefly, normal saline was added at a ratio of 1:9 to weighted, frozen intestinal tissue. The homogenates were then centrifuged to obtain the supernatant, after which the protein concentration was measured using the Bradford method. Finally, the Na^+^-K^+^-ATPase activity was determined by measuring the level of Pi using malachite green dye and expressed as micromoles per milligram protein.

### 2.8. Analysis of the Intestinal Villi Integrity 

Scanning electron microscopic (SEM) was employed to observe the integrity of the jejunum villi, as previously described [24]. After the intestinal contents were cleaned with saline, the jejunum was immersed in 2% glutaraldehyde (0.1 mol/L PBS) for 2 d. Transverse slices of each intestinal segment (2 mm × 2 mm) were prepared and immersed in a 2.5% osmium tetroxide aqueous solution for 3 d in a thermostatic chamber (20 °C) for tissue maceration and subsequently treated in an ultrasonic bath for 10 min to remove the epithelium from the intestinal villi. The samples were then stained with a 1% tannic acid solution and 1% osmium tetroxide for 2 h, respectively, dehydrated in a graded ethanol series (70%, 80%, 90%, and 95% for 30 min each and three times at 100% for 30 min), and dried in a critical point dryer. The dried samples were mounted on aluminum stubs with double-sided carbon tape, coated with platinum–palladium using a magnetron sputtering coater (MSP-10, Vacuum Device Co., Ltd., Ibaraki, Japan), and observed using SEM (Phenom-World PW-100-016, Eindhoven, Netherlands).

### 2.9. Quantification of the Cytokine and TJ Protein Expression in the Intestinal Tissue

The intestinal segments (100 mg) were homogenized with 900 μL of cell lysis buffer containing a protease inhibitor cocktail over ice for 30 min. The samples were centrifuged at 12,000 g for 10 min, after which the total protein lysate was collected to quantitatively detect the different cytokines and TJ proteins, including IL-4, IL-10, IL-18, IL-22, TNF-α, IFN-γ, mucin-2, β-defensins, claudin-1, occludin, and ZO-1, using an ELISA kit according to the instruction of the manufacturer.

### 2.10. 16S rDNA Gene Sequencing of the Gut Microbiota

Here, 16S rRNA gene amplicon sequencing was performed using the Illumina MiSeq platform (Novogene Bioinformatics Technology Co., Ltd., Beijing, China), as described in a previous report [25], to determine the primer sequences targeting the V4 hypervariable region of the bacterial 16S rRNA genes (515F = 5′-GTGCCAGCMGCCGCGGTAA-3′, and 806R = 5′-GGACTACNNGGGTATCTAAT-3′). The bioinformatic sequencing data were analyzed using the Quantitative Insights into Microbial Ecology (QIIME) software. Briefly, the raw sample data were filtered and spliced to obtain high-quality clean reads. The effective tags were then clustered into operational taxonomic units (OTU) at an identity threshold of 97% similarity. Taxonomic ranks were assigned to representative OTU sequences using Ribosomal Database Project (RDP) Classifier v 2.2 [26]. Finally, species annotations were created for the representative sequences of each OTU, while the corresponding species information and species-based abundance distribution were obtained. Furthermore, the OTUs were analyzed for relative abundance to obtain the microbial richness and diversity between the different groups.

### 2.11. Statistical Analysis 

The data were shown as means ± SD. The statistical analysis was performed via one-way ANOVA and Duncan’s multiple-comparison test. These calculations were carried out using the SPSS software (version 20.0, IBF Inc., Chicago, IL, USA). Differences of *p* < 0.05 were considered statistically significant.

## 3. Results

### 3.1. Clinical Symptoms and Anatomic Observations of the Allergic Mice

**Figure 2:**
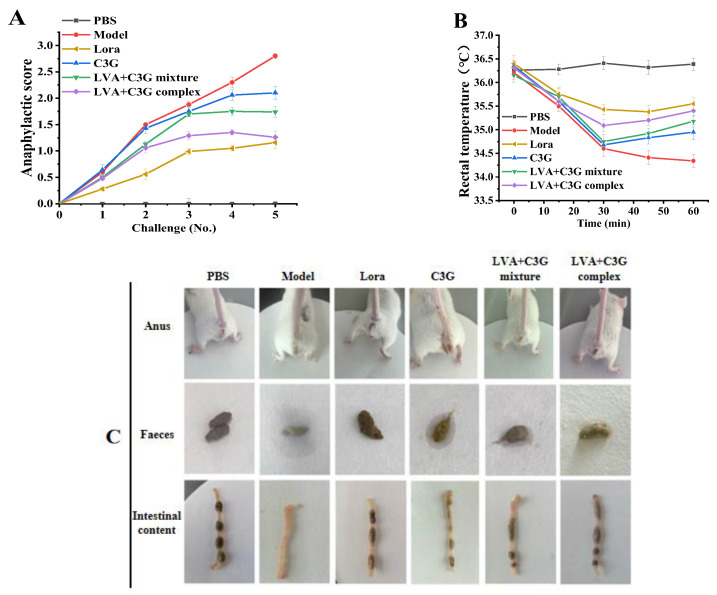

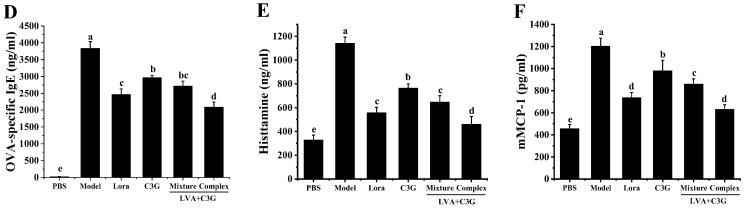
The effect of the C3G-associated samples on the allergic response. (**A**) The scores of the anaphylactic symptoms. (**B**) The rectal temperature of the mice 0 to 1 h after the last challenge. (**C**) Anal, fecal, and intestinal content observations. (**D**) Serum OVA-specific IgE. (**E**) Serum histamine. (**F**) Serum mMCP-. Significant differences (*p* < 0.05) are identified by different letters.

As shown in Figure 2A–C, the clinical symptoms of the mice were examined. The normal mice (PBS group) displayed strong mobility and a good mental state, as well as smooth, shiny fur. Clean anuses, granular feces, lumpy intestinal content, and stable rectal temperatures (averaging 36.3 °C) were also observed. Contrarily, after the fifth OVA oral challenge, the sensitized mice exhibited reduced motor activity, lethargy, shivering, curled hair, and other allergic symptoms (the anaphylactic score was up to 2.8 ± 0.05, while the rectal temperature decreased to 34.3 °C). Clear signs of diarrhea included porridge-like droppings around the anus, loose, unformed stools, and fluid intestinal content. However, the C3G-associated samples inhibited these allergic symptoms in sensitized mice to varying degrees. In particular, animals treated with the LVA + C3G complex displayed noticeably better symptomatology than the animals who received the LVA + C3G mixture or C3G treatment. The inhibitory effect of the LVA + C3G complex on allergic reactions was close to that of the positive Lora control. 

The serum OVA-specific IgE, histamine, and mMCP-1 levels were determined to further investigate the effect of C3G, the LVA + C3G mixture, and the LVA + C3G complex on anaphylaxis in OVA-induced allergic mice. As expected, the serum OVA-specific IgE, histamine, and mMCP-1 levels in the model group were exceedingly high, reaching 3833.53 ± 203.04 ng/mL, 1141.52 ± 51.61 ng/mL, and 1202.74 ± 73.01 pg/mL, respectively (Figure 2D). Contrarily, the serum OVA-specific IgE levels were significantly (*p* < 0.05) reduced when the animals were treated with C3G, the LVA + C3G mixture, or the LVA + C3G complex. The LVA + C3G complex performed the best. Compared with the Lora group (35.7% reduction), a 45.6% reduction in the OVA-specific IgE level was observed in the LVA + C3G complex group. Similarly, the LVA + C3G complex displayed a better (*p* < 0.05) inhibitory effect on the serum histamine and mMCP-1 levels than both C3G and the LVA + C3G mixture (Figure 2E,F). After LVA + C3G complex administration, the serum histamine and mMCP-1 levels decreased significantly to 460.87 ± 65.17 ng/mL and 630.83 ± 41.77 pg/mL, respectively. These results indicated that LVA-based self-assembly could enhance the effect of C3G in alleviating allergic symptoms.

### 3.2. Observation of the Physical Barrier Function of the Intestinal Epithelium

**Figure 3:**
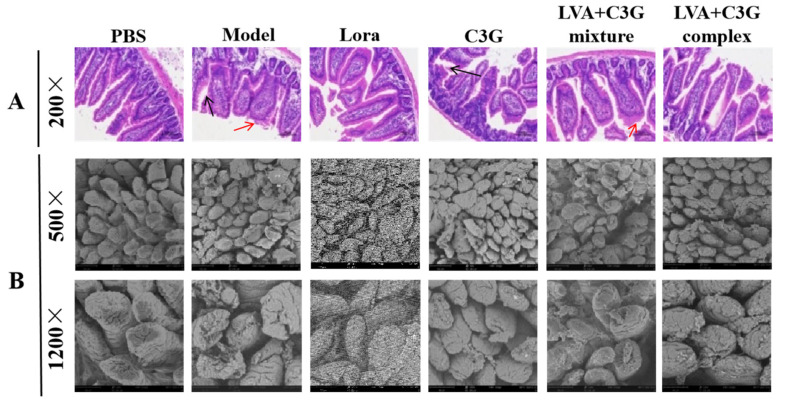

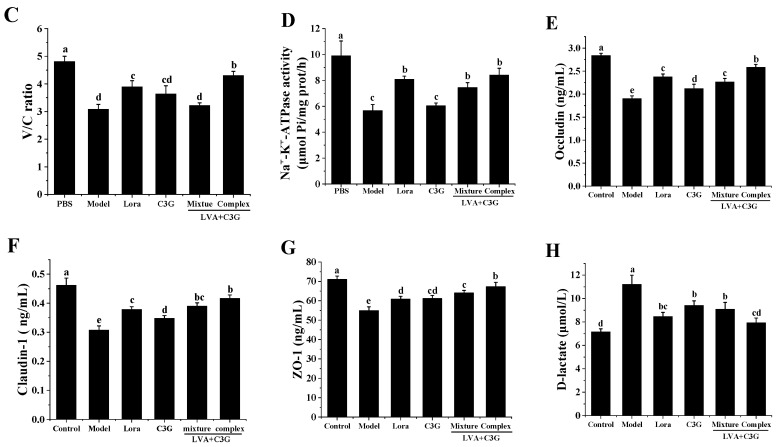
The effect of the C3G-associated samples on the physical barrier function of the intestinal epithelium. (**A**) H&E-stained cecum sections. Red arrows indicate mucosal atrophy and edema, and black arrows indicate crypt hyperplasia. (**B**) SEM observation of the cecum villi. (**C**) The V/C ratio. (**D**) Intestinal Na+-K+-ATPase activity. (**E**) Occludin expression. (**F**) Claudin-1 expression. (**G**) ZO-1 expression. (**H**) Serum D-lactic acid. Significant differences (*p* < 0.05) are identified by different letters.

Food allergic reactions, especially with diarrhea, are often accompanied by intestinal inflammation. Therefore, this work employed H&E staining and SEM to examine the intestinal inflammation in OVA-induced FA mice. The H&E staining results showed several signs of morphological damage in the model group, including mucosal atrophy and edema, irregularly shaped villi, and crypt hyperplasia (Figure 3A). Moreover, SEM revealed ruptured jejunal villi surfaces and increased local damage in OVA-allergic mice (Figure 3B). However, this morphological damage was effectively attenuated in the C3G-associated samples, especially in the LVA + C3G complex treatment group. The villus length to crypt depth (V/C) ratio was calculated to quantify the altered jejunal morphology. The V/C ratio of the jejuna in the model group (3.08 ± 0.18) was significantly (*p* < 0.05) lower than in the PBS group (4.81 ± 0.18) (Figure 3C). After C3G, LVA + C3G mixture, and LVA + C3G complex administration, the V/C ratio increased to 3.64 ± 0.30, 3.22 ± 0.10, and 4.30 ± 0.15, respectively. In addition, the activity of the Na^+^-K^+^-ATPase was detected. As depicted in Figure 3D, the Na^+^-K^+^-ATPase activity in the model group was significantly (*p* < 0.05) lower than in the PBS group. The LVA + C3G complex exhibited a distinctly better ameliorative effect in restoring the Na^+^-K^+^-ATPase activity than C3G and the LVA + C3G mixture.

### 3.3. Observation of the Chemical Barrier Function of the Intestinal Epithelium

**Figure 4:**
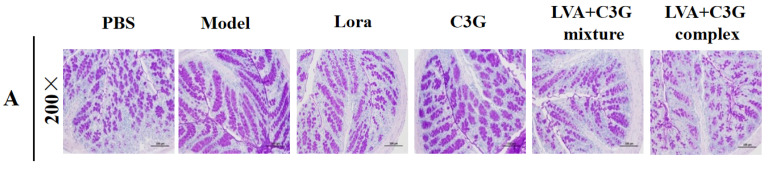

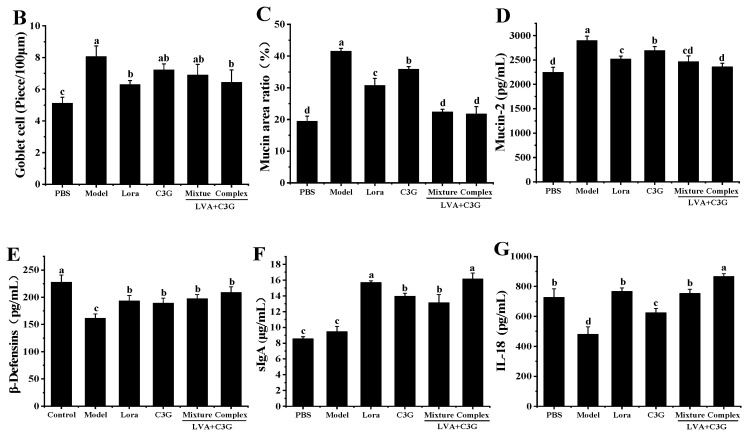
The effect of the C3G-associated samples on the chemical barrier function of the intestinal epithelium. (**A**) Photomicrographs of a PAS-stained colon section. (**B**) Goblet cells per unit length (100 μm). (**C**) The mucin area ratio of the mucus layer. (**D**) The mucin-2 levels in the mucus layer. (**E**,**F**) The β-defensin and sIgA levels in the mucus layer. (**G**) The intestinal IL-18 levels. Significant differences (*p* < 0.05) are identified by different letters.

Since the intestinal mucus layer is essential in maintaining intestinal barrier homeostasis, this study investigates the related changes. As shown in Figure 4A–C, the PAS staining results showed an abnormal increase in goblets cell and mucin secretion in the model group, which were 0.58-fold and 1.13-fold, respectively, higher than in the PBS group. However, the C3G-associated samples, especially the LVA + C3G complex, markedly reversed these abnormal changes. Mucin-2, the main gelatin-forming protein of the intestinal mucus layer, was also examined, showing that the LVA + C3G mixture and LVA + C3G complex restored the mucin-2 content to normal levels (Figure 4D). 

The C3G-associated samples also enhanced β-defensin and sIgA secretion in the mucus layer. After C3G, LVA + C3G mixture, and LVA + C3G complex administration, the β-defensin levels in the model group increased from 161.41 to 189.32 pg/mL, 197.46 pg/mL, and 208.84 pg/mL, respectively (Figure 4E). Additionally, compared with the PBS group, increased sIgA levels of 63.08%, 53.50%, and 88.69% were observed in the C3G, LVA + C3G mixture, and LVA + C3G complex groups (Figure 4F). Compared with the model group, the level of IL-18, an important cytokine that maintains intestinal mucosal homeostasis, increased by 30.08%, 57.00%, and 80.41% in C3G, LVA + C3G mixture, and LVA + C3G complex groups, respectively (Figure 4G).

### 3.4. Regulation of the Intestinal Immune-Related Cytokines

**Figure 5:**
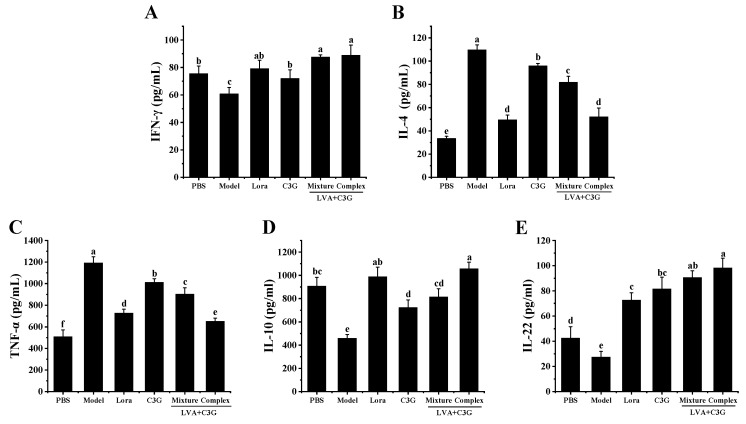
The effect of the C3G-associated samples on the intestinal immune-related cytokines. (**A**) IFN-γ. (**B**) IL-4. (**C**) TNF-α. (**D**) IL-10. (**E**) IL-22. Significant differences (*p* < 0.05) are identified by different letters.

An imbalance in T helper (Th)1/Th2 cytokine secretion ultimately leads to IgE-mediated allergic reactions. Therefore, the secretion of the signature IFN-γ and IL-4 Th1/Th2 cytokines was investigated. As expected, the model group displayed significantly reduced (*p* < 0.05) IFN-γ levels (by 19.48%), while IL-4 was substantially (*p* < 0.05) higher (by 228.58%), indicating OVA-stimulated differentiation of antigen-specific Th cells was skewed to the Th2 phenotype (Figure 5A,B). Simultaneously, a significant (*p* < 0.05) rise in the allergic inflammation cytokine, TNF-α (increased by 1.35-fold) was observed, in contrast to an obvious (*p* < 0.05) decrease in the anti-inflammatory cytokine, IL-10 (reduced by 49.42%) (Figure 5C,D). Moreover, the IL-22 levels produced by the lamina propria lymphocytes declined by 49.42% (Figure 5E). The dysregulation of the intestinal immune-related cytokines was effectively (*p* < 0.05) inhibited by the C3G-associated samples, especially the LVA + C3G complex. The Th1/Th2 cytokine secretion imbalance was restored in the allergic mice of the LVA + C3G complex group, accompanied by a 46.22% increase in IFN-γ and a 52.53% decrease in IL-4. In addition, TNF-α decreased remarkably (*p* < 0.01) from 1192.19 ± 56.11 pg/mL (model group) to 651.20 ± 29.70 pg/mL. Furthermore, the IL-10 (from 458.93 ± 31.90 pg/mL to 1056.60 ± 56.80 pg/mL) and IL-22 (from 27.44 ± 4.54 pg/mL to 98.26 ± 7.72 pg/mL) increased significantly (*p* < 0.01) compared to the model group.

### 3.5. Analysis of the Composition of the Flora

**Figure 6:**
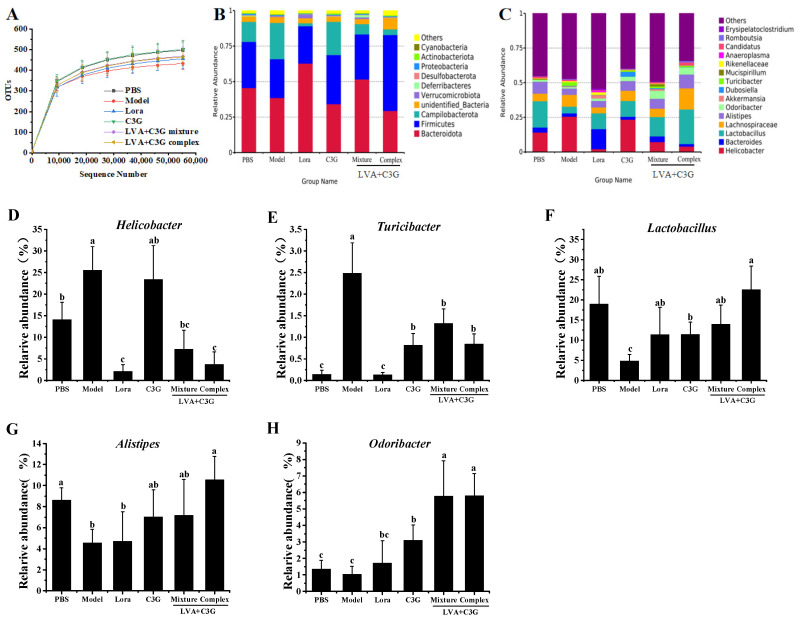
The effect of the C3G-associated samples on microbial composition. (**A**) The rarefaction curves of the OTUs of each group. (**B**) The composition of the flora at the phylum level. (**C**) The composition of the flora at the genus level. (**D**–**H**) The relative abundance of the selected bacteria at the genus level ((**D**) Helicobacter. (**E**) Turicibacter. (**F**) Lactobacillus. (**G**) Alistipes. (**H**) Odoribacter). Significant differences (*p* < 0.05) are identified by different letters.

The gut microbial changes in the different groups were further analyzed via 16S rDNA high-throughput sequencing. As shown in Figure 6A, the rarefaction curve of each sample tended to be flat, suggesting that the number of identified OTUs reached saturation as the number of sequences increased, validating the sequencing results. The diversity, evenness, and richness of the bacterial community were statistically determined from sequencing data based on Shannon, Simpson, abundance coverage-based estimator (ACE) and Chao1 indices (Table S1). Shannon and Simpson indices showed that there were no significant differences in intestinal microbial diversity and evenness among all groups. ACE and Chao1 indices indicated lower species richness in the model group than those in the other groups. At the phylum level, *Bacteroidota* (45.57%), *Firmicutes* (32.50%), and *Campilobacterota* (14.10%) were the dominant phyla in the PBS group (Figure 6B). After five OVA gavages, the bacterial community abundance exhibited distinct differences, displaying reduced *Bacteroidota* (38.53%) and *Firmicutes* (27.37%) levels and increased *Campilobacterota* (25.54%). After Lora and C3G-associated administration, the total relative abundance of *Bacteroidota* and *Firmicutes* increased while *Campilobacterota* decreased. The *Bacteroidota* (51.64%) and *Firmicutes* (31.67%) abundance in the LVA + C3G mixture group was close to that of the normal mice (PBS group). 

At the genus level, the dominant bacteria in the PBS group were Helicobacter (14.10%), *Bacteroides* (3.65%), *Lactobacillus* (18.95%), *Lachnospiraceae* (5.43%), and *Alistipes* (8.62%) (Figure 6C). In the sensitized mice, the pathogenic *Helicobacter* and *Turicibacter* bacteria increased 0.81-fold and 16.79-fold, respectively, while *Lactobacillus* and *Alistipes* decreased by 74.53% and 46.98%, respectively (Figure 6D–G). As expected, the LVA + C3G complex exhibited the best rehabilitative effect on the bacterial community. After administration of the LVA + C3G complex, *Helicobacter* and *Turicibacter* decreased to 3.70% and 0.84%, respectively, while *Lactobacillus* and *Alistipes* displayed a respective increase to 22.52% and 10.52%. The abundance of *Odoribacter* increased from 1.03% in the model group to 5.81% in the LVA + C3G complex group (Figure 6H).

## 4. Discussion

Dietary polyphenols reportedly exhibit extensive anti-allergic properties [1,27]. However, polyphenols, especially anthocyanins, are unstable in adverse gastrointestinal environments, severely restricting their biological effect in vivo [8]. Studies have shown that polysaccharide-based encapsulation protects polyphenols from adverse conditions in the upper gastrointestinal tract, delivering as much polyphenol as possible to the intestine for an enhanced biological effect [7,9,10]. This work assessed the anti-allergic activity of the LVA + C3G complex in an OVA-induced mouse model. As expected, the LVA + C3G complex performed better than free C3G in alleviating allergic reactions and regulating intestinal microecological disruption.

FA is an immunologically adverse reaction, inducing a range of IgE-mediated disorders, including anaphylaxis, decreased body temperature, and adverse gastrointestinal reactions [28]. This study showed that all interventions with oral C3G-associated samples alleviated OVA-induced allergic symptoms, reduced histamine, and mMCP-1 levels, inhibited OVA-specific IgE, and improved inflammatory intestinal epithelial damage to some extent. Interestingly, the physical mixture of LVA and C3G (LVA + C3G mixture) was more effective in alleviating allergic reactions than C3G alone, probably due to the fact that LVA also has immunomodulatory activity and its synergy with C3G increased the effect of alleviating FA. A previous study reported that alginate inhibited OVA-induced allergic reactions in mice [29], which supports our view. More interestingly, the LVA + C3G complex was more successful than LVA + C3G mixture in alleviating allergic reactions, suggesting that it is the LVA-based encapsulation that further enhances the effect of C3G in alleviating FA. The efficacy of the LVA + C3G complex in alleviating allergic reactions was close to that of the positive control, Lora, which has been shown to inhibit the development of diarrhea and decrease the severity of symptom scores at a dose of 20 mg/kg [30]. 

A defective intestinal epithelial barrier increases intestinal permeability and plays an essential role in FA development [31]. When the intestinal barrier is impaired, food allergens are excessively exposed to the mucosal immune system, promoting acute and profound inflammatory immune responses [23,31,32]. Therefore, as an important physical barrier, the intestinal epithelium plays a critical role in improving tolerance to potential orally ingested food allergens. The TJ proteins between the IECs are crucial for maintaining intestinal barrier integrity [33]. In this study, C3G-associated administration enhanced occludin, claudin-1, and ZO-1 expression in the OVA-induced allergic mice. This indicated that the C3G-associated samples encouraged the TJs in the IECs to improve mucosal integrity and enhance intestinal epithelial barrier function. Furthermore, the serum D-lactate levels were reduced, which was associated with improved intestinal mucosal barrier functionality [34]. These results showed that the C3G-associated samples enhanced intestinal barrier function by regulating TJ proteins to ameliorate FA. The findings were consistent with a previous study, indicating that baicalein helped attenuate FA by regulating the TJs in the epithelium [35]. The study results also emphasized that the LVA + C3G complex performed better than the LVA + C3G mixture and C3G in enhancing intestinal epithelial barrier functionality.

In addition, the mucus layer covering the gut epithelium is critical to the epithelial defense system, constituting the first line of defense against enteric dietary antigens and invading pathogens [14]. The mucus barrier is a repository of antimicrobial peptides (AMPs) and immunoglobulins, representing one of the main drivers of intestinal homeostasis [1]. The sIgA produced by plasma cells in the lamina propria can be directly transported by IECs and is actively secreted into the intestinal mucus layer to block toxins and pathogens from adhering to the intestinal epithelium [34]. Moreover, sIgA reportedly neutralizes allergens and helps to limit the entry of allergens into the lamina propria, restricting the inflammatory response [36]. In addition, the IL-18 produced by IECs acts as a pleiotropic cytokine that induces enterocytes to generate more AMPs to destroy pathogens liking *Salmonella typhimurium* [37]. As primary AMPs, β-defensins are constitutively secreted from IECs, affecting the gut mucosal defense system [34]. In this study, the C3G-associated samples alleviated the abnormal mucus secretion caused by FA while increasing the sIgA and β-defensin levels in the mucus layer. Therefore, the oral administration of the C3G-associated samples reinforced the defenses of the intestinal mucus layer. A more protective biochemical barrier was created for the intestine via the spatial segregation of luminal antigens and bacteria from direct interaction with the intestinal epithelium.

FAs are caused by an imbalance in the Th1/Th2 immune responses [23]. As an important Th2 cytokine, IL-4 stimulates B cell differentiation and IgE production, inducing the degranulation of mast cells and aggravating allergic reactions. However, the IFN-γ secreted by Th1 cells inhibits IgE secretion and eosinophil production to alleviate allergic reactions [38]. In this study, the IFN-γ level increased, and the IL-4 level decreased in sensitized mice after treatment with the C3G-associated samples, indicating that the Th1/Th2 immune balance was directed toward Th1 dominance. These results demonstrated that the C3G-associated samples displayed the same effect as other polyphenols, reducing the imbalance of the Th1/Th2 immune response to relieve FA symptoms in the entire immune system and gastrointestinal reactions [39]. Furthermore, the excessive production of TNF-α, an essential pro-inflammatory cytokine, can induce cell apoptosis, disrupt intestinal epithelial integrity, and weaken intestinal barrier functionality [40]. IL-10 is necessary to maintain the integrity and homeostasis of intestinal epithelia. It can limit inflammation development and facilitate the tissue healing process caused by the inflammatory response [31,41]. In addition, as an immunosuppressive cytokine, IL-10 can differentiate and maintain regulatory T cells, which is crucial for immune tolerance and immune response reinforcement [23,42]. IL-22 is secreted by lamina propria lymphocytes and is essential for mucosal homeostasis regulation [37]. This study observed a decrease in the TNF-α concentrations and an increase in the IL-10 and IL-22 concentrations in the gut after administration of the C3G-associated treatment, which was consistent with enhanced intestinal barrier integrity. Therefore, treatment with the C3G-associated samples maintained intestinal immune homeostasis by regulating the intestinal immune-related cytokines, contributing to FA alleviation.

Mounting evidence indicates that gut microbiota plays a central role in regulating food-mediated allergic reactions [1]. Although many studies support the evidence that polyphenols can modulate the development of FA, research linking the role of polyphenols in regulating FA to the gut microbiota remains limited. It has been reported that changes in the composition of the gut microbiota are associated with the development of FA [43]. Previous studies showed that the abundance of *Firmicutes* and *Clostridia* were reduced in patients with milk allergies, and the *Firmicutes* and *Ruminococcaceae* were lower in mice with OVA-induced FA [6]. This study revealed a significant decrease in the abundance of *Firmicutes* in OVA-sensitized mice, which was associated with an aggravated response to food antigens [5]. However, treatment with the C3G-associated samples, particularly the LVA + C3G complex, significantly increased *Firmicutes* abundance. Additionally, the C3G-associated sample treatments substantially increased the proportion of beneficial bacteria, *Lactobacillus* and *Odoribacter*, in the OVA-sensitized mice. The crucial role of commensal microbiota in either the susceptibility to or protection against FA is attributed to its effect on the physical and immunological barrier function of the intestinal epithelium [43]. Studies have shown that many *Lactobacillus* strains can regulate intestinal homeostasis [44]. For example, *Lactobacillus casei* LC122 increased TJ- and AMP-related gene expression, effectively inhibiting permeability and enhancing barrier function in cells and animals [45]. The beneficial effects of *Odoribacter* are primarily attributed to the production of SCFAs, such as butyrate that promotes the proliferation and differentiation of Treg cells and TJ assembly, consequently enhancing the integrity of the intestinal barrier [46]. Therefore, it is reasonable to infer that the increased relative abundance of *Lactobacillus* and *Odoribacter* may somehow play a role in the ameliorative effect of the C3G-associated samples on FA. Additionally, the decline in the relative abundance of the pathogenic *Helicobacter* and *Turicibacter* bacteria may contribute to FA amelioration by the C3G-associated samples. As the primary destructive bacteria in the intestinal immune system, *Helicobacter* is associated with the emergence of FA [47]. The genus *Turicibacter* belongs to the class *Erysipelotrichi*, which is associated with intestinal inflammation and penetrability [48]. The results of this study suggest that the C3G-associated samples, especially the LVA + C3G complex, are beneficial in modulating gut microbiota, helping to alleviate FAs. Although the mechanism remains unclear, these observations may raise questions worthy of further exploration.

## 5. Conclusions

In conclusion, the present study confirms that LVA-based self-assembly enhances the alleviating effect of C3G on OVA-induced FA. The LVA + C3G complex effectively reduces OVA-induced allergic symptoms, lowers histamine and mMCP-1 levels, inhibits OVA-specific IgE, and improves inflammatory-induced intestinal epithelial injury. Intestinal barrier function enhancement, immune homeostasis maintenance, and compositional modulation of the intestinal flora may represent some of the contributing mechanisms responsible for attenuating allergic responses. Overall, the LVA + C3G complex may provide a novel means of treating OVA-induced allergic responses and may have therapeutic potential for allergic disease induced by food allergens.

## Data Availability

No new data were created or analyzed in this study. Data sharing is not applicable to this article.

## Supplementary Materials

The following supporting information can be downloaded at: https://www.mdpi.com/article/10.3390/foods11111542/s1, Table S1: Alpha diversity analysis of intestinal microflora in each group.
