# Supplementary material for: Amelioration of Ovalbumin-Induced Food Allergy in Mice by Targeted Rectal and Colonic Delivery of Cyanidin-3-O-Glucoside
Source: Foods. 2022 May 24;11(11):1542. doi: 10.3390/foods11111542 (PMC9180400; doi:10.3390/foods11111542)
Supplement: Supplementary file 1 [file foods-11-01542-s001.zip › foods-1707076-Sypplementary.pdf]

Table S1. Alpha diversity analysis of intestinal microflora in each group.

Group	Shannon index	Simpson index	ACE index	Chao1 index
PBS	5.88 ± 0.43[a]	0.95 ± 0.02[a]	492.15 ± 44.67[ab]	491.77 ± 41.86[ab]
Model	5.90 ± 0.39[a]	0.94 ± 0.04[a]	430.07± 29.81[b]	430.09 ± 27.84[b]
Lroa	5.79 ± 0.58[a]	0.95 ± 0.04[a]	453.70 ± 51.89[ab]	452.10 ± 51.03[ab]
C3G	5.55 ± 0.79[a]	0.90 ± 0.09[a]	496.70 ± 53.46[a]	495.06 ± 51.14[a]
LVA+C3G Mixture	5.73 ± 0.66[a]	0.94 ± 0.04[a]	479.13 ± 34.84[ab]	478.20 ± 32.66[ab]
LVA+C3G Complex	5.80 ± 0.41[a]	0.95 ± 0.03[a]	460.60 ± 49.63[ab]	460.28 ± 49.00[ab]

Diversity estimates: Shannon and Simpson indices. Species richness estimates: ACE and Chao1 indices. C3G, cyanidin-3-O-glucoside; LVA, low-viscosity sodium alginate; ACE, abundance coverage-based estimator. The data were shown as means ± SD. Significant differences ($P < 0.05$) between groups are identified by different letters.
